# Defective Generation and Maturation of Dendritic Cells from Monocytes in Colorectal Cancer Patients during the Course of Disease

**DOI:** 10.3390/ijms141122022

**Published:** 2013-11-07

**Authors:** Giulia Orsini, Annalisa Legitimo, Alessandra Failli, Paola Ferrari, Andrea Nicolini, Roberto Spisni, Paolo Miccoli, Rita Consolini

**Affiliations:** 1 Laboratory of Immunology, Department of Clinical and Experimental Medicine, University of Pisa, via Roma, 67, Pisa 56126, Italy; E-Mails: a.legitimo@ao-pisa.toscana.it (A.L.); faillialessandra@gmail.com (A.F.); rita.consolini@med.unipi.it (R.C.); 2 Department of Clinical and Experimental Medicine, Azienda Ospedaliero-Universitaria Pisana (AOUP), via Roma, 67, Pisa 56126, Italy; E-Mail: p.ferrari@ao-pisa.toscana.it; 3 Department of Clinical and Experimental Medicine, Section Medical Oncology, University of Pisa, via Roma, 67, Pisa 56126, Italy; E-Mail: andrea.nicolini@med.unipi.it; 4 Department of Surgery, Molecular, Medical and Critical Area Pathology, University of Pisa, via Paradisa, 2, Pisa 56126, Italy; E-Mails: roberto.spisni@med.unipi.it (R.S.) paolo.miccoli@med.unipi.it (P.M.)

**Keywords:** monocyte-derived dendritic cells, colorectal cancer, immunosuppression

## Abstract

Colorectal cancer (CRC) is the second-leading cause of cancer-related deaths in Western countries. Today, the role of the host’s immune system in controlling the progression and spread of solid tumors is broadly established. Tumor immunosurveillance escape mechanisms, such as those involving dendritic cells (DCs), the most important antigen-presenting cells, are likewise recognized processes involved in cancer. The present study evaluates the ability of CRC patients to generate DCs *in vitro* from circulating monocytes at both pre- and post-operative timepoints; the results are correlated with the stage of disease to shed light on the systemic immune statuses of CRC patients. Our data showed that patients’ DCs had lower co-stimulatory molecule expression and were less able to present antigens to allogeneic T cells compared to healthy controls’ (HC) DCs. Furthermore altered cytokine secretion, such as increased IL-10 and reduced IL-12 and TNF-α, was observed. At the post-operative timepoints we observed a recovery of the patients’ ability to generate immature DCs, compared to HCs, but the maturational capacity remained affected. Our study conclusively highlights the persistently impaired *in vitro* generation of fully mature and functional DCs, which appears to be more altered during advanced stages. This work sheds light on a dendritic cell-based tumor immune escape mechanism that could be useful for the development of more effective immunotherapeutic strategies.

## 1. Introduction

Currently the host immune system’s role in controlling the progression and spread of solid tumors has been broadly established [1]. Colorectal cancer (CRC) is among the most common malignancies in Western countries and has a high mortality rate [2]; furthermore, CRC has long been considered scarcely immunogenic, but several findings have recently shown that anti-tumor immune responses might occur in these patients [3].

Among the immune cell populations, tumor-infiltrating T lymphocytes (TILs) are reportedly associated with improved clinical outcomes and survival in CRC patients, regardless of the tumor stage [4]. Moreover, an intra-tumoral correlation has also been demonstrated between the numbers of T and dendritic cells (DCs) [5]; the latter are the most important antigen-presenting cells (APCs) and have recently been associated with better clinical outcomes in CRC patients [6]. Myeloid dendritic cells are derived from blood monocytes (MoDCs). Initially, immature DCs (iDCs) are produced and can recognize and capture antigens in peripheral tissues, after which they develop into mature DCs (mDCs) upon antigen uptake. These mDCs then migrate to the lymph nodes where, once activated, they can efficiently prime both CD4^+^ and CD8^+^-specific T cell responses. Final maturation is determined by pro-inflammatory cytokines that induce the upregulation of co-stimulatory molecules such as CD40, CD80, MHC class II (HLA-DR) molecules, and the maturation marker CD83, on the DC surface; the presence of these molecules is fundamental to correct antigen presentation to lymphocytes [7]. Pro-inflammatory cytokines secreted by mature DCs, including IL-12 and TNF-α, are also important for effective T cell proliferation and activation, whereas anti-inflammatory cytokines, such as IL-10 and TGF-β1 have been shown to inhibit T cell immune activity and induce Treg cells [8]. Fully mature DCs have been shown to drive effective anti-tumor cytotoxic T cell immune responses in appropriate cytokine microenvironments [9], whereas antigen presentation by immature DCs in an inadequate cytokine milieu leads to the inhibition of T cell functions [10,11]. Quantitative and functional impairments of circulating DCs have been widely observed in several types of cancer [12–15], and these represent a tumor-escape mechanism employed by cancer cells to elude host immunosurveillance [16,17]. Several studies have also demonstrated impairments of DC differentiation from CD14^+^ monocyte precursors and of iDC maturation in cancer patients, both of which lead to dysfunctional DCs that cannot present antigens and thus induce tolerogenicity [18–24]. The infiltration rates and local distribution of DCs in CRC tissues have been investigated broadly [5,6,25–30]. Conversely, few studies have been performed on the abilities of these patients to generate fully functional DCs at a circulating level [18,31,32].

Therefore the aim of the present study was to investigate the abilities of circulating monocytes from early- and advanced-stage CRC patients to generate DCs, to evaluate both the phenotypic and functional features of *in vitro*-generated iDCs and mDCs, and to correlate the results with the disease stage. Furthermore, we monitored the patients at specific timepoints after surgical resection to identify the DC status throughout the disease course.

## 2. Results and Discussion

### 2.1. Patient Characteristics

A total of 23 CRC patients were enrolled in this study. The average age (±SEM) of the patients was 68.48 (±2.29) years (median, 70 years; Table 1). One patient died immediately after surgery, and 1 patient presented with a distant metastasis at the time of diagnosis (stage IV); as this subject underwent continuous chemotherapeutic treatment after surgical resection, we could not collect any other blood samples after the pre-surgical sample. Fourteen of the 23 patients had minimal follow-up durations of approximately 2 months after the baseline blood sample collection, whereas the other patients were lost after surgical resection. A third blood sample was obtained from 11 of the 14 patients who underwent the second blood collection. Additionally, 45% of these patients underwent adjuvant chemotherapy before sampling; one of these patients presented with pulmonary metastasis during chemotherapy and was therefore excluded from the third timepoint analysis. Finally, 8 patients underwent the last sampling; 3 of these patients were in stage I, 4 in stage II and 1 in stage III.

### 2.2. Phenotypic and Functional Characterizations of MoDCs

#### 2.2.1. Pre-Surgery

**Figure 1:**
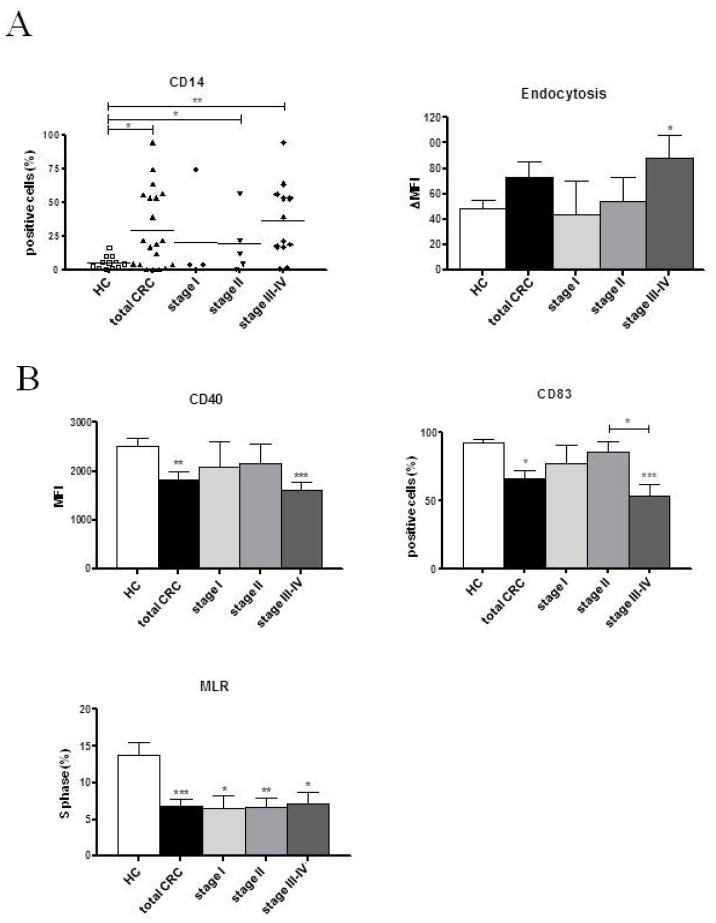

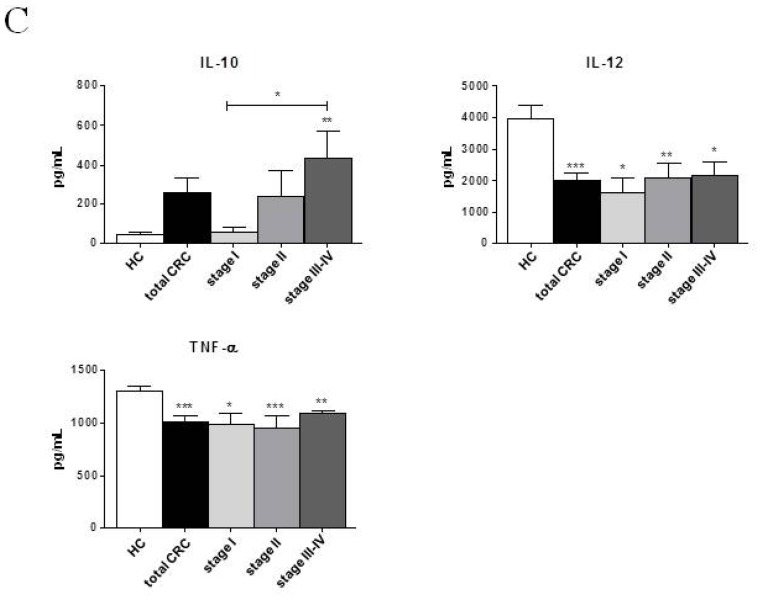
Phenotypic and functional characteristics of MoDCs derived from CRC patients at the pre-surgery timepoint. Evaluations were performed at the (**A**) sixth and (**B**,**C**) seventh days (after LPS addition) of culture. CRC patients, *n* = 23; stage I, *n* = 5; stage II, *n* = 6; stage III–IV, *n* = 12; HCs *n* = 14. Data are shown as the means ± SEM. *p < 0.05; **p < 0.01; ***p < 0.001. Statistical significance is in comparison to HCs, except where indicated. MoDCs: monocyte-derived dendritic cells; LPS: lipopolysaccharide; HC: healthy controls; CRC: colorectal cancer; MLR: mixed lymphocyte reaction; MFI: mean fluorescence intensity. The *n* indicates the number of cases analyzed.

Colorectal cancer patients exhibited an impaired capacity to generate immature DCs from blood monocytes compared to HCs, as shown by the significantly higher percentage of CD14 expressing cells (29.42% ± 6.16% *vs*. 5.51% ± 1.38%, respectively; *p* < 0.05; Figure 1A). This result could be attributed to the more advanced-stage CRC subjects (stages II and III–IV: 19.33% ± 9.90% and 36.50% ± 8.15%, respectively *vs*. 5.51% ± 1.38%; *p* < 0.05 and *p* < 0.01, respectively), as the stage I patients were not significantly different from the controls. Endocytic activity was significantly higher in stage III–IV patients than controls (ΔMFI: 88.52 ± 17.08 *vs*. 48.83 ± 5.78; *p* < 0.05).

Phenotypic analyses performed after LPS stimulation revealed lower expression levels of the co-stimulatory marker CD40 on CRC patient cells compared to HC cells (CD40 MFI: 1828.00 ± 171.40 *vs*. 2526.37 ± 147.80, *p* < 0.01; Figure 1B); this was mainly attributable to stage III–IV subjects (CD40 MFI: 1065.94 ± 180.60 *vs*. 2526.37 ± 147.80, *p* < 0.001). Patients also displayed significantly lower expression levels of the maturational marker CD83 (positive cells: 65.87% ± 6.29% *vs*. 92.84% ± 1.88%; *p* < 0.05). Notably, a significant reduction in this antigen was observed in stage III–IV subjects, compared to HCs and stage II patients (positive cells: 53.63% ± 8.28% *vs*. 92.84% ± 1.88% and 85.83% ± 7.44%, respectively; *p* < 0.001 and *p* < 0.05, respectively). Moreover, CRC patients showed a significantly reduced ability to stimulate allogeneic T lymphocyte proliferation, compared to HCs (S phase: 6.86% ± 0.93% *vs*. 13.78% ± 1.73%, respectively; *p* < 0.01). Similar results were obtained when each single stage was compared to the controls (Figure 1B). An evaluation of the levels of secreted cytokines in the culture supernatants was performed on day 7 (Figure 1C). Stage III–IV patients showed significantly higher levels of IL-10 secretion than either controls or stage I subjects (453.30 ± 138.10 pg/mL *v*s. 45.05 ± 11.59 pg/mL and 58.48 ± 22.21 pg/mL, respectively; *p* < 0.01 and *p* < 0.05, respectively). Significant reductions in IL-12 and TNF-α secretion were observed in patients, compared to HCs (IL-12: 1978.93 ± 249.80 pg/mL *vs*. 3972.11 ± 443.90 pg/mL; *p* < 0.001 and TNF-α: 1011.61 ± 51.30 pg/mL *vs*. 1307.47 ± 43.63 pg/mL; *p* < 0.001, respectively). Overlapping results were obtained for patients at all stages of disease. However, IL-6 production was comparable between the patients and controls, whereas TGF-β1 was substantially absent in both groups (data not shown).

**Figure 2:**
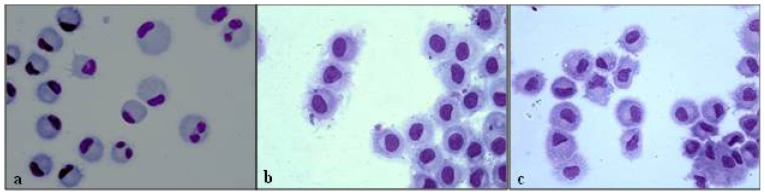
MoDCs at the pre-surgery timepoint. Cells were obtained at the seventh day of culture (after LPS addition) from (**a**) a healthy control; (**b**) a stage II-CRC patient; and (**c**) a stage III-CRC patient. The morphological analysis was performed by light microscopy (magnification, 100×) on MoDCs previously stained with May-Grünwald-Giemsa. A representative experiment is shown. MoDCs: monocyte-derived dendritic cells; LPS: lipopolysaccharide; CRC: colorectal cancer.

A morphological analysis performed on LPS-matured DCs from 5 stage II (Figure 2b) and 5 stage III (Figure 2c) patients showed a centrally positioned nuclei, less-defined cytoplasm and shorter dendrites than cells from healthy donors (Figure 2a); all these features indicated a more immature phenotype.

#### 2.2.2. Follow-up

The comparison between the first and second timepoints was performed with paired data, including only CRC patients who underwent both blood samplings. Both timepoints were also compared with the healthy control data.

At the second sampling, taken at a maximum of two months after surgery, the percentage of CD14-expressing iDCs in patients was reduced, compared to the first sampling, whereas endocytosis remained significantly higher in stage III subjects than in HCs (Table 2).

After LPS stimulation, CD40 expression remained significantly lower in patients than in controls, whereas CD80 expression became significantly lower in patients at the second timepoint, compared to healthy donors. The increase in HLA-DR expression after LPS stimulation was significantly lower than the previous sampling. However, CD83 expression did not significantly differ between patients and HCs. When comparing the second and first timepoint data, we observed a general slight increase in maturational markers expression, except for CD80 expression (Table 2). Moreover, at the second timepoint the ability to stimulate allogeneic T lymphocyte proliferation remained significantly lower in patients than in HCs. Additionally, the comparison with the first timepoint data showed a slight decrease in the MLR capability. Similar results were obtained when stages I and II were analyzed separately. At this timepoint, the IL-10, IL-12 and TNF-α levels were altered in the CRC subjects, compared to the controls. In particular, the IL-10 levels were significantly higher in the total, stage II and III patients, compared to the HCs; in the stage III patients, this significant increase persisted from the first pre-operative sampling. In contrast, the levels of secreted IL-12 and TNF-α were significantly lower in CRC subjects than in healthy donors, similar to the results obtained at the first timepoint. The comparison between the second and first timepoint data revealed a slight increase in the secretion of all investigated cytokines. Notably, IL-12 production significantly increased in the total patient group, compared to the same patients at the pre-operative sampling (Table 2).

Data collected at the third timepoint were examined in two different types of analysis. The first compared the controls with total CRC patients and in two patient subgroups, according to whether the patients received chemotherapeutic treatment.

**Figure 3:**
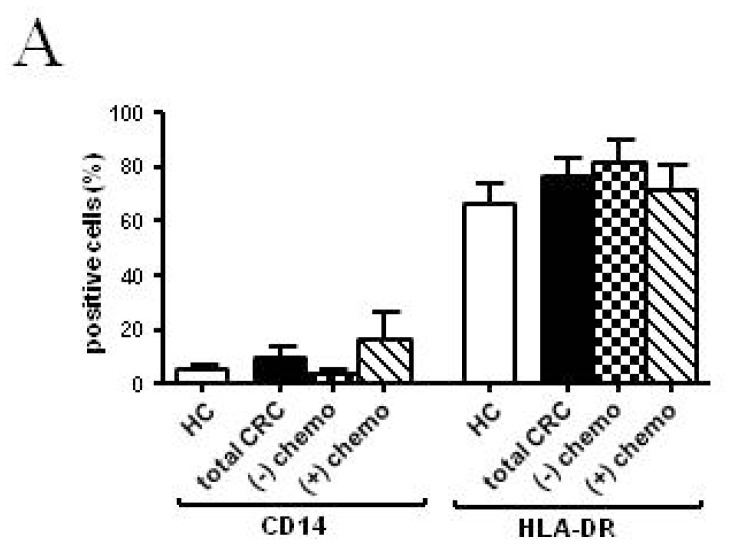

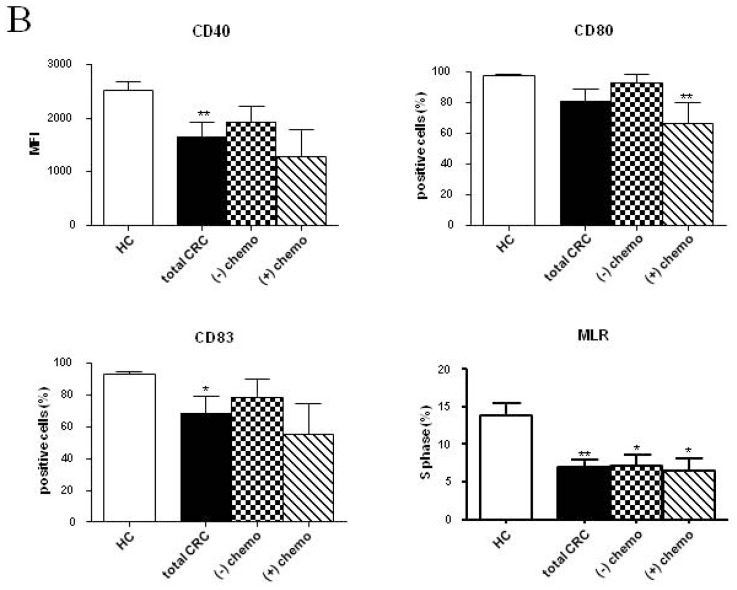
Phenotypic and functional characteristics of MoDs. A comparison of the total, chemotherapy-treated and untreated CRC patients and healthy controls at the third timepoint: a. Evaluations were performed at the (**A**) sixth and (**B**) seventh days of culture (after LPS addiction). CRC patients, *n* = 11; untreated patients, *n* = 6; chemotherapy-treated patients, *n* = 5; HCs, *n* = 14. Data are shown as the means ± SEM. *p < 0.05; **p < 0.01. Statistical significance is in comparison to HCs, except where indicated. MoDCs: monocyte-derived dendritic cells; LPS: lipopolysaccharide; HC: healthy controls; CRC: colorectal cancer; MLR: mixed lymphocyte reaction; MFI: mean fluorescence intensity; the *n* indicates the number of cases analyzed.

The ability to generate immature DCs from patients was comparable to that from controls, as shown by the similar percentages of CD14 and HLA-DR-expressing cells (Figure 3A). In contrast, maturational phenotypic features were somewhat altered in the total patient or chemotherapy-treated patient groups, compared to the controls (CD40 MFI: 1641.82 ± 277.40 *vs*. 2526.37 ± 147.80, total CRC *vs*. HCs; *p* < 0.01 and CD80 positive cells: 66.26% ± 13.24% *vs*. 97.34% ± 0.72%, treated-patients *vs*. HCs; *p* < 0.01. CD83 positive cells: 68.36% ± 10.58% *vs*. 92.84% ± 1.88%, total CRC *vs*. HCs; *p* < 0.05; Figure 3B). Functional analysis revealed that mature DCs from patients were significantly less able to stimulate allogeneic T cell proliferation than those from healthy donors (S phase: 6.90% ± 1.00%, *p* < 0.01, 7.18% ± 1.42% and 6.55% ± 1.60%, *p* < 0.05, *vs*. 13.78% ±1.73%, in total CRC, untreated and chemotherapy-treated patients, respectively). Additionally, IL-10, IL-12 and TNF-α secretion were significantly impaired in all patient groups, compared to the controls (Table 3).

An additional third timepoint analysis was performed by retrospectively dividing the data obtained at the previous two blood samplings according to whether the patients received chemotherapeutic treatment or not.

As shown in Table 4, our results showed that the chemotherapy-treated patients had progressively lower percentages of CD14-expressing cells than were observed at the two previous timepoints. Furthermore, at the pre-operative timepoint, these subjects also had evidently higher percentages of CD14-expressing cells than did the untreated subjects. This observation could be due to the more advanced disease severity at diagnosis in chemotherapy-treated patients. We also observed no significant difference in the abilities of the chemotherapy-treated and untreated CRC subjects to generate mature DCs at the three timepoints. In contrast, a comparison between these patients and HCs showed significant differences, especially in the ability to obtain fully mature DCs, as shown by the impaired functional capacities (Table 4).

### 2.3. Discussion

Despite recent advances in surgery and radio- and chemotherapeutic regimens, colorectal cancer is the second-leading cause of cancer-related deaths in Western countries [2]. Recently, several studies have demonstrated that the host immune system status in the tumor microenvironment constitutes an important prognostic factor for patient survival [33,34]. Over the last few years several studies have focused on dendritic cells, the major antigen-presenting cells, which play a pivotal role in the induction of anti-tumor cytotoxic immune responses and further link the adaptive and innate immune systems [9]. Tumor cells, especially those in early stages of cancer development, have been shown to construct several immune escape mechanisms that involve DCs, to elude host immune system control [11]. Interestingly, a recent study by Scarlett *et al*. showed that, in a mouse model of ovarian cancer, early-stage tumor growth is controlled and inhibited by the recruitment of immunostimulatory DCs to the tumor microenvironment and the subsequent induction of T cell anti-tumor immunity. Aggressive tumor growth coincides with phenotypic and functional immunosuppressive changes to these DCs [35]. Moreover, the numbers of circulating and tumor-infiltrating DCs were markedly reduced in CRC patients [6,15,17]. As several immunotherapeutic vaccine trials include the use of *ex vivo* tumor antigen-loaded autologous DCs that are re-infused into the patients, investigations of the ability to generate fully mature and functional DCs from circulating patient monocytes are highly important [36]. Unlike other tumors, the effects of CRC on patients’ systemic immunological statuses have been scarcely investigated [18,31,32].

Our data shows the impaired *in vitro* differentiation of patient monocytes into immature DCs, compared to those from healthy controls. The persistent CD14 expression on patient DCs might suggest a more marked differentiation of monocytes towards the macrophage rather than the dendritic component [37]; this could also explain the higher endocytic activity observed in patients, compared to controls [38,39]. However, the evaluation of FITC-dextran uptake by DCs did not provide insight into the ability of these cells to process internalized antigens, which might not be as effective as uptake, thus inducing altered DC maturation. Macrophages can play a dual role in cancer progression depending on their phenotype; most tumor-infiltrating macrophages present a M2 phenotype, which promotes immunosuppression by secreting IL-10 and plays a role in tumor neovascularization and metastasis by producing pro-angiogenic factors such as VEGF-A, IL-8 and basic fibroblastic growth factor (bFGF) [40]. Moreover, a recent study showed that CD14^+^ DCs induced by the addition of IL-10 during MoDC maturation exhibited a tolerogenic macrophage-like phenotype [41].

Furthermore, CRC patient DCs showed a reduced ability to present antigens to allogeneic T lymphocytes and to stimulate proliferation, together with a significantly altered co-stimulatory molecule expression compared to controls. Accordingly, we observed a markedly immunosuppressive cytokine profile in the patient DCs, characterized by increased IL-10, and reduced IL-12 and TNF-α secretion. Similarly, Michielsen *et al.* previously showed that CRC-conditioned medium negatively influenced DC maturation and IL-12 secretion, while augmenting IL-10 secretion [42].

Interleukin-10 is a well-known anti-inflammatory factor that inhibits DC differentiation and maturation *in vitro* and consequently suppresses T cell stimulation [11]. Additionally, it can determine immune system anergy and/or tolerogenity by promoting the DC-mediated polarization of naïve T lymphocytes to the Th2 and/or type-1 regulatory T cell subsets [43,44]. In fact, IL-10 is among the most important cytokines released by tumor cells to suppress host immune function [17]. In contrast, IL-12 and TNF-α are the major pro-inflammatory cytokines secreted by DCs, to induce the Th1 polarization of naive T cells and cytotoxic immune responses [45,46]; furthermore, secretion of these cytokines is negatively modulated by immunosuppressive factors such as IL-10 [8]. This altered cytokine profile could account for the DC impairments and tumor-related immune deficiencies in patients. Therefore, our results show that CRC exerts negative effects on DC generation and maturation at a systemic level and that both of these deficiencies appear to correlate with the disease stage. These observations agree with those of previous studies on different tumor types [20–22,24,47,48]. Conversely, Gabrilovich *et al.* showed that DCs isolated from breast cancer patient blood samples are impaired with regard to phenotypic features and *in vitro* antigen presentation, whereas DCs generated *in vitro* from blood precursors in the same patient samples are totally functional; the authors concluded that defective DC function can be at least partially overcome by removing the tumor immunosuppressive factors and generating DCs *in vitro* [49]. This finding is in contrast to our data. Furthermore, we demonstrated that DCs generated from CRC patients *in vitro* release immunosuppressive cytokines that could also exert autocrine effects. Additionally, our and Gabrilovich’s studies differ in terms of the tumor type and methodologies, which could account for the different results obtained.

At the post-operative timepoint we observed a recovery of the ability to generate DCs from CRC patients, compared to controls, although tumor resection did not improve the phenotypic and functional characteristics of these mature DCs. This result partially agrees with a previous study by Bellik *et al*., who established that after surgical resection, CRC patients recovered the ability to generate MoDCs, compared to untreated CRC subjects. In contrast, the same authors also observed that these MoDCs expressed increased levels of maturational markers [32]. Another study performed on prostate cancer patients demonstrated that tumor resection restored the mature DC phenotypic profile, compared to healthy subjects [19]. We suggest that the observed impairments in mature DC function are partially attributable to the altered *in vitro* cytokine production, as these cytokines would therefore not exert their autocrine/paracrine effects. However, the complex mechanisms responsible for the prolonged developmental impairments in DCs from patient monocytes remain unclear.

Our results showed that chemotherapeutic treatment did not affect the patients’ ability to generate DCs when compared to both untreated patients and healthy controls. The maturational process was significantly impaired at both the phenotypic and functional levels, regardless of the treatment received. These observations suggest that the ability to generate fully mature and functional DCs from CRC patients remains impaired even at six- to ten months after surgical tumor resection. Similarly, at twelve months after surgery, the DC maturational process in CRC patients included some phenotypic and cytokine alterations. At this final sampling, a comparison of the CRC patients at the four timepoints was performed by dividing the patients into those who received chemotherapy and those who did not, although the number of subjects belonging to the first group was too small to allow statistical analyses. We observed that at all four timepoints, the percentage of CD14-expressing cells in the treated patients remained higher than in the untreated ones. The maturational phenotypic characteristics tended to decrease in a linear manner in untreated patients, but increased in treated patients after tumor removal surgery. Cytokine production was comparable between the two CRC groups, except for IL-10, the secretion of which was markedly higher in treated patients than in untreated ones. No patients studied until this timepoint developed relapses or metastases, but long-term clinical evaluations such as the 5-year overall and disease-free survival rates, along with a broader cohort of patients, could be useful to clarify whether the DC impairment effectively persists after longer time intervals.

## 3. Experimental Section

### 3.1. Study Population

In this study we enrolled 23 CRC patients with newly histologically confirmed diagnoses of colorectal adenocarcinoma; the patients included 17 males and 6 females between 42 and 86 years of age. A total of 5 patients had stage I disease (T1-2N0M0), 6 had stage II (T3-4N0M0), 11 had stage III (any T, N1-2M0) and 1 had stage IV (any T, any N, M1). We divided the populations into groups based on clinical stage; because of the small number of stage IV patients, we combined stages III and IV when performing statistical analyses. None of the patients has previous histories of colorectal cancer or neoplastic diseases; furthermore the CRC patients had not been treated with neoadjuvant chemotherapy. Colorectal cancer patients who underwent adjuvant chemotherapy received the following regimen: 5-fluorouracil (5FU) + leucovorin (LV) + oxaliplatinum.

As controls, we enrolled a population of 14 age-matched healthy subjects (HCs) without any histories or diagnoses of neoplastic diseases. Patients or healthy subjects with immune system diseases such as autoinflammatory, rheumatic and congenital immunodeficiencies, viral and/or bacterial infections, hematological diseases, or diabetes and those who were treated with immunosuppressive drugs were excluded from the study. The baseline clinical characteristics of the CRC patients and healthy controls are described in Table 1.

From each CRC patient, the first blood sample was drawn at the baseline, before surgical resection (first or pre-operative timepoint); the second was collected at the first follow-up medical examination, approximately two months after surgery and before adjuvant chemo- and/or radiotherapy (second timepoint); the third was drawn approximately six- to ten months after surgical resection and/or two months after the end of adjuvant chemotherapy (third timepoint), and the last (fourth timepoint) was drawn at a minimum of twelve months after surgery and/or six months after the end of adjuvant chemotherapy, both during routine follow-up examinations.

The study was conducted with the approval of the local Ethical Committee. All CRC patients and healthy volunteers gave their informed consent for inclusion in this study.

### 3.2. Generation of Dendritic Cells

Human heparinized peripheral blood samples were drawn from CRC patients and healthy donors, and the peripheral blood mononuclear cells (PBMCs) were separated by Ficoll-Paque density gradient centrifugation (Lympholyte-H, Cedarlane Laboratories Ltd, Burlington, ON, Canada). CD14-positive cells were purified from the PBMCs by positive selection with the MIDIMACS technique, using monoclonal mouse anti-human CD14-coated microbeads (Miltenyi Biotec GmbH, Bergisch Gladbach, Germany) according to the manufacturer’s instructions and as previously described [50]. The monocytes were resuspended in RPMI-1640 (Gibco Laboratories, Grand Island, NY, USA) supplemented with 10% of fetal bovine serum, 2 mM l-glutamine (Euroclone SpA, Pavia, Italy), 100 μg/mL streptomycin (Sigma-Aldrich Co., St. Louis, MO, USA), 100 IU/mL penicillin (Sigma-Aldrich Co., St. Louis, MO, USA) at a concentration of 1 × 10^6^ cells/mL and cultured at 37 °C and 5% CO_2_ for 6 days in a 24-well tissue culture plate in the presence of 50 ng/mL of recombinant human granulocyte-macrophage colony-stimulating factor (GM-CSF; PeproTech Inc., Rocky Hill, NJ, USA) and 20 ng/mL of recombinant human interleukin-4 (IL-4; PeproTech Inc., Rocky Hill, NJ, USA). The samples were fed with fresh cytokines every 3 days. To induce DC maturation, immature DCs received 100 ng/mL of bacterial lipopolysaccharide (LPS; Sigma-Aldrich Co., St. Louis, MO, USA) on day 6 for a 24-h period.

### 3.3. Morphological Evaluation

To evaluate the cultured cell morphology, cytospin slides were prepared by cytocentrifuging 100 μL of a 5 × 10^4^ cells/mL suspension at 800 *g* for 10 min (Shandon Cytospin-2 Centrifuge, GMI Inc., Ramsey, MN, USA). The slides were air-dried and fixed with methanol (Carlo Erba Reagenti SpA, Milan, Italy), stained with May-Grünwald-Giemsa (Carlo Erba Reagenti SpA, Milan, Italy) and then examined by light microscopy (Olympus Corporation, Tokyo, Japan).

### 3.4. Phenotypic Analysis

The harvested cells were labeled with the following mouse monoclonal antibodies for cell-surface staining: anti-CD14, anti-human leucocyte antigen D-related (HLA-DR) (Immunotools, Friesoythe, Germany), anti-CD40, anti-CD80, and anti-CD83 (Immunotech SAS, Marseille, France); the antibodies were conjugated with FITC (fluorescein isothiocyanate) or PE (phycoerythrin). The cells were washed in a saline solution that contained PBS, 0.2% FBS and 0.05% sodium azide (NaN_3_; Sigma-Aldrich Co., St. Louis, MO, USA). A negative control (cells without any monoclonal antibodies) was included for each sample to exclude cell autofluorescence. Cell acquisition and data analysis were performed on an Epics-XL (Beckman Coulter Inc., Miami, FL, USA) with Expo32 Software (Beckman Coulter Inc., Miami, FL, USA). 5000 events were acquired for each sample. The DCs were gated on the basis of their light-scattering properties, and dead cells were excluded from the analysis. The results were expressed as the percentage of positive cells or the mean fluorescence intensity (MFI) of the positive cells.

### 3.5. Phagocytosis Assay

Mannose receptor-mediated phagocytosis was measured by cytofluorimetry as the cellular uptake of FITC-dextran (Sigma-Aldrich Co., St. Louis, MO, USA). Approximately 1 × 10^6^ immature DCs were incubated in RPMI-1640 with 10% FBS and 2 mg/mL FITC-dextran for 60 min at 37 °C or on ice (negative control to exclude unspecific external binding). After the incubation, the cells were washed twice with PBS to remove excess dextran and fixed with cold 1% formaldehyde (Bio Optica Milano SpA, Milan, Italy). 5000 events were acquired for each sample. The DCs were gated on the basis of their light-scattering properties, and dead cells were excluded from the analysis. The quantification of phagocytosis was expressed as the difference in MFI (ΔMFI) between cell fractions of the same sample that were incubated at 37 °C and at 0 °C.

### 3.6. Allogeneic T Cell Proliferation Assay

For the primary mixed lymphocyte reaction (MLR) assay, a T cell population isolated via the immunomagnetic method from healthy donors after the Ficoll-separation of peripheral blood mononuclear cells, was used as responder cells to test the stimulatory capacities of DCs. Graduated numbers of 7-day-cultured-DCs were added to 2 × 10^5^ allogeneic responder T cells per well of a 96-well round-bottomed cell culture plate and were maintained at 37 °C and 5% CO_2_. Control cultures with responder or stimulator cells only were assessed to evaluate the background proliferation. All experiments were performed in triplicate. After 6 days of culture, the cells were collected, counted, diluted to 1 × 10^6^ cells/mL, fixed with 70% ethanol, and stored at −20 °C for 24 h. Proliferation according to the cell-cycle S phase was assessed by DNA staining with propidium iodide (PI, 20 μg/mL; eBioscience Inc., San Diego, CA, USA). Data acquisition and analysis were performed on an Epics-XL flow cytometer with MultiCycle software.

### 3.7. Cytokine Measurements

CRC and control cell culture supernatants were harvested on day 7 to analyze the following cytokines: IL-6, IL-12, IL-10 and TNF-α. Briefly, cell suspensions were collected and centrifuged and the supernatants were filtered through 0.2-μm pore filters (International Pbi SpA, Milan, Italy) and stored at −80 °C. Cytokine concentrations were measured via the immunoenzymatic method (ELISA), according to the manufacturer’s instructions.

### 3.8. Statistical Analysis

Statistical analyses were performed with GraphPad Prism software (GraphPad Software Inc., La Jolla, CA, USA). The results were expressed as the means ± standard errors of the mean (SEM). Significant differences between CRC patients and healthy subjects were determined with two-tailed unpaired Student’s *t*-tests. *p*-values < 0.05 were considered statistically significant.

## 4. Conclusions

Our work highlights a significant and persistent impairment in *in vitro* generation of fully mature and functional DCs from CRC patients, which appears more pronounced at advanced stages of the disease. Furthermore, this study contributes to the understanding of the biological interactions between the host immune system and the tumor by elucidating a tumor-escape mechanism employed by CRC cells. Further studies are needed to determine whether DC phenotypic and functional status could be considered a useful parameter to critically address tailored therapeutic strategies when selecting possible cancer patients for DC-based immunotherapy.

## Figures and Tables

**Table 1:** Baseline characteristics of CRC patients and healthy controls.

	HC	CRC			
		
		Total CRC	Stage I	Stage II	Stage III–IV
**Number of subjects (*n*)**	14	23	5 (21.74)	6 (26.09)	12 (52.17)

**Age (years)**					

Mean	59.79 ± 4.83	68.48 ± 2.29	70.00 ± 2.41	69.67 ± 6.94	67.25 ± 2.82
Range	29–85	42–86	63–77	42–86	47–80

**Sex**					

**M**	9	17	4	4	9
**F**	5	6	1	2	3

**Tumor location:**	N/A				

Right side of colon		9 (39.13)	2 (40.00)	3 (50.00)	4 (33.33)
Left side of colon		4 (17.39)	3 (60.00)	1 (16.67)	0
Transverse colon		1 (4.35)	0	0	1 (8.33)
**Rectum**		9 (39.13)	0	2 (33.33)	7 (58.33)

**Adjuvant chemotherapy**	N/A				

Lost cases ‡		7 (30.43)	1 (20.00)	1 (16.67)	5 (41.67)
Yes		8 (34.78)	0	1 (16.67)	7 (58.33)
**No**		8 (34.78)	4 (80.00)	4 (66.67)	0

**WBC (×10^3^/μL)**	6.80 ± 0.34	6.93 ± 0.36	5.86 ± 0.27	7.26 ± 0.84	7.22 ± 0.51

**MNC (×10^3^/μL)**	2.54 ± 0.13	2.21 ± 0.12 *	2.21 ± 0.16	2.39 ± 0.31	2.12 ± 0.17

Data are shown as the mean values ± SEM; percentage values are in parentheses;

* *p* < 0.05; statistical significance is in comparison to HC;

‡ CRC patients lost throughout the experimental period;

CRC: colorectal cancer; HC: healthy controls; WBC: white blood cells; MNC: mononuclear cells; N/A: not available.

**Table 2:** Comparison of data from healthy controls and post- and pre-surgical colorectal cancer patients.

	HC (*n* = 14)	Total CRC		Stage I		Stage II		Stage III	
		
		1° tp (*n* = 14)	2° tp (*n* = 14)	1° tp (*n* = 4)	2° tp (*n* = 4)	1° tp (*n* = 5)	2° tp (*n* = 5)	1°tp (*n* = 5)	2° tp (*n* = 5)
**DC +6**									

CD14 (%)	5.51 ± 1.38	21.45 ± 6.53 *	13.47 ± 4.38	3.39 ± 0.95	7.31 ± 5.96	19.00 ± 10.02	12.91 ± 6.45	38.84 ± 12.18 ***	18.97 ± 9.80
HLA-DR (%)	66.15 ± 7.57	71.21 ± 6.49	67.73 ± 5.40	64.30 ± 14.02	73.57 ± 11.03	88.54 ± 4.74	64.24 ± 8.47	61.50 ± 10.72	66.55 ± 10.52
Endocytosis (ΔMFI)	48.83 ± 5.78	79.40 ± 18.44	63.67 ± 10.28	57.47 ± 28.90	57.60 ± 12.20	60.28 ± 19.34	55.76 ± 19.96	112.00 ± 34.50 *	75.74 ± 14.04 *

**DC +7**									

CD40 (MFI)	2526.37 ± 147.80	1802.22 ± 253.70 *	1898.17 ± 152.30 **	2482.10 ± 469.80	1792.77 ± 323.10	1845.64 ± 504.90	2172.12 ± 144.70	1350.86 ± 244.90 ***	1687.46 ± 311.70 *
CD80 (MFI)	137.41 ± 7.26	123.96 ± 21.85	112.65 ± 9.07 *	153.95 ± 15.65	116.63 ± 16.26	116.90 ± 43.53	120.80 ± 12.11	108.10 ± 22.50	99.48 ± 20.97
CD83 (%)	92.84 ± 1.88	65.64 ± 8.67	81.56 ± 6.14	79.86 ± 18.39	84.28 ± 12.84	73.69 ± 12.02	90.98 ± 1.95	49.06 ± 15.02 *	70.50 ± 13.68
HLA-DR (MFI)	267.70 ± 34.13	300.30 ± 34.55	212.20 ± 24.17 †	388.20 ± 81.11	240.30 ± 62.05	282.20 ± 22.29	206.10 ± 29.21	260.30 ± 68.32	178.60 ± 29.90
S phase (%)	13.78 ± 1.73	7.75 ± 1.22 **	7.21 ± 0.96 **	7.97 ± 1.34	5.05 ± 2.33 *	4.93 ± 0.92 **	7.13 ± 1.23 *	10.43 ± 2.59	9.00 ± 1.13

**Cytokines +7**									

IL-6 (pg/mL)	281.89 ± 16.37	253.42 ± 15.58	284.16 ± 10.52	223.13 ± 46.49	279.93 ± 20.07	264.43 ± 20.55	290.55 ± 20.70	265.13 ± 20.17	280.95 ± 19.05
IL-10 (pg/mL)	45.05 ± 11.59	239.76 ± 92.31	320.16 ± 86.52 *	48.53 ± 28.10	254.63 ± 198.30	187.65 ± 152.20	228.55 ± 113.70 *	435.28 ± 178.20 **	460.90 ± 159.80 *
IL-12 (pg/mL)	3972.11 ± 443.90	2064.82 ± 281.10 ***	2751.64 ± 104.30 **,†	1863.33 ± 596.50 *	2455.67 ± 368.00 *	1896.75 ± 519.60 *	2827.00 ± 25.33 *	2384.00± 471.90 *	2898.25 ± 20.69 *
TNF-α (pg/mL)	1307.47 ± 43.63	991.67 ± 64.52 ***	1064.21 ± 24.27 ***	949.93 ± 137.80 *	1019.77 ± 92.78 *	916.48 ± 146.40 **	1069.57 ± 15.10 **	1098.20 ± 33.50 *	1092.18 ± 10.15 *
TGF-β1 (pg/mL)	6.18 ± 0.28	5.81 ± 0.60	5.89 ± 0.58	7.00 ± 0.87	4.93 ± 1.44	6.13 ± 0.72	5.20 ± 0.41	4.60 ± 1.24	7.30 ± 0.90

Data are shown as the mean values ± SEM; Statistical significance is in comparison to healthy controls, unless otherwise indicated;

* *p* < 0.05;

** *p* < 0.01;

*** *p* < 0.001;

† *p* <0 .05;

2° time-point *vs*. 1° time-point; The *n* in brackets indicates the number of cases analyzed; HC: healthy controls; CRC: colorectal cancer; DC: dendritic cells; tp: timepoint.

**Table 3:** Cytokine determination: a comparison of total, chemotherapy-treated and untreated patients and healthy controls at the third timepoint.

	HC (*n* = 14)	Total CRC (*n* = 11)	No Chemotherapy (*n* = 6)	Plus Chemotherapy (*n* = 5)
**IL-10 (pg/mL)**	45.05 ± 11.59	289.40 ± 84.10 *	271.62 ± 118.30 *	311.63 ± 137.10 *
**IL-12 (pg/mL)**	3972.11 ± 443.90	2542.87 ± 164.10 **	2535.16 ± 244.30 *	2552.50 ± 249.10 *
**TNF-α (pg/mL)**	1307.47 ± 43.63	1008.46 ± 52.32 ***	1028.70 ± 21.00 ***	983.15 ± 123.70 *

Data are shown as the mean values ± SEM; Statistical significance is in comparison to healthy controls, unless otherwise indicated;

* *p* < 0.05;

** *p* < 0.01;

*** *p* < 0.001;

HC: healthy controls; CRC: colorectal cancer; The *n* in brackets indicates the number of cases analyzed.

**Table 4:** Comparison of data from healthy controls and CRC patients among the three timepoints.

	HC (*n* = 14)	CRC					
		
		No chemotherapy (*n* = 6)			Plus chemotherapy (*n* = 5)		

DC +6		1° time point	2° time point	3° time point	1° time point	2° time point	3° time point
CD14 (%)	5.51 ± 1.38	8.57 ± 3.89	10.42 ± 4.23	3.72 ± 1.54	32.09 ± 13.47	25.94 ± 11.68	16.64 ± 9.92
HLA-DR (%)	66.15 ± 7.57	70.31 ± 16.73	65.88 ± 5.98	81.62 ± 8.14	61.06 ± 14.38	66.10 ± 8.76	71.00 ± 9.39
endocytosis (ΔMFI)	48.83 ± 5.78	68.58 ± 20.20	77.92 ± 11.30	58.38 ± 28.91	112.1 ± 45.78	54.08 ± 20.41	66.70 ± 21.38

**DC +7**							

CD40 (MFI)	2526.37 ± 147.80	2858.77 ± 267.40	1794.06 ± 130.80 *,†	1925.86 ± 288.20	1549.30 ± 193.80 **	1977.97 ± 300.10	1286.78 ± 497.70
CD80 (%)	97.34 ± 0.72	98.97 ± 0.56	89.00 ± 8.16	92.59 ± 5.72	92.92 ± 2.07	85.58 ± 12.55	66.26 ± 13.24 **
CD83 (%)	92.84 ± 1.88	98.66 ± 0.53 *	80.43 ± 12.15	78.71 ± 11.37	66.99 ± 14.46	77.87 ± 17.77	55.41 ± 18.87
HLA-DR (MFI)	267.70 ± 34.13	279.80 ± 24.21	225.00 ± 51.51	229.90 ± 44.96	257.00 ± 48.43	202.40 ± 32.99	187.10 ± 65.34
S phase (%)	13.78 ± 1.73	5.80 ± 1.31 *	4.75 ± 1.51 **	6.20 ± 1.32 *	9.70 ± 2.39	9.15 ± 1.44	6.55 ± 1.60 *

**Cytokines +7**							

IL-6 (pg/mL)	281.90 ± 16.37	223.40 ± 46.51	310.50 ± 10.53	279.20 ± 20.80	265.10 ± 20.17	264.00 ± 20.86	280.50 ± 19.50
IL-10 (pg/mL)	45.05 ± 11.59	28.53 ± 9.59	260.00 ± 159.90	213.10 ± 132.50	558.90 ± 153.10 **	321.30 ± 175.50 *	311.60 ± 137.10 *
IL-12 (pg/mL)	3972.11 ± 443.90	1758.00 ± 692.50 *	2833.33 ± 41.28 *	2313.27 ± 370.50 *	2852.00 ± 23.39 *	2872.00 ± 20.95 *	2552.50 ± 249.10 *
TNF-α (pg/mL)	1307.47 ± 43.63	884.10 ± 203.50 *	1106.57 ± 17.19 *	1032.97 ± 27.06 **	1108.22 ± 26.63 *	1089.03 ± 9.35 **	983.15 ± 123.70 *
TGF-β1 (pg/mL)	6.18 ± 0.28	6.87 ± 0.87	5.87 ± 1.16	5.73 ± 2.28	4.76 ± 1.22	6.40 ± 1.07	6.40 ± 1.82

Data are shown as the mean values ± SEM; Statistical significance is in comparison to healthy controls, unless otherwise indicated;

* *p* < 0.05;

** *p* < 0.01;

† *p* < 0.05;

2° time point *vs*. 1° time point; HC: healthy controls; CRC: colorectal cancer; DC: dendritic cells; the *n* in brackets indicates the number of cases analyzed.
